# Antimicrobial Susceptibility and Resistance Genes in *Streptococcus uberis* Isolated from Bovine Mastitis in the Czech Republic

**DOI:** 10.3390/antibiotics12101527

**Published:** 2023-10-11

**Authors:** Monika Zouharova, Katerina Nedbalcova, Katarina Matiaskova, Petr Slama, Jan Matiasovic

**Affiliations:** 1 Department of Infectious Diseases and Preventive Medicine, Veterinary Research Institute, 621 00 Brno, Czech Republic; katerina.nedbalcova@vri.cz (K.N.); katarina.matiaskova@vri.cz (K.M.); jan.matiasovic@vri.cz (J.M.); 2 Laboratory of Animal Immunology and Biotechnology, Department of Animal Morphology, Physiology and Genetics, Faculty of AgriSciences, Mendel University, 613 00 Brno, Czech Republic; petr.slama@mendelu.cz

**Keywords:** *Streptococcus uberis*, bovine mastitis, antimicrobial resistence, antimicrobial resistance gene, intramammary infection, whole-genome sequencing

## Abstract

*Streptococcus uberis* is one of the most important causative agents of mastitis and is a common reason for the use of antimicrobials in dairy cows. In this study, we assessed the antimicrobial susceptibility of 667 *S. uberis* isolates originating from 216 Czech dairy farms collected between 2019 and 2023 using the broth microdilution method. We tested 140 of the isolates for the presence of antimicrobial genes using whole-genome sequencing and evaluated their relationship with phenotypic resistance. *Streptococcus uberis* isolates showed high levels of resistance to tetracycline (59%), followed by streptomycin (38%) and clindamycin (29%). Although all of the isolates were susceptible to beta-lactams, a relatively high percentage of intermediately susceptible isolates was recorded for ampicillin (44%) and penicillin (18%). The isolates were mainly resistant to tetracycline alone (31.3%); the second most frequent occurrence of the phenotypic profile was simultaneous resistance to tetracycline, streptomycin, and clindamycin (16.6%). The occurrence of antibiotic resistance genes did not always match the phenotypic results; in total, 36.8% of isolates that possessed the *ant(6)-Ia* gene did not show phenotypic resistance to streptomycin. To a lesser extent, silent genes were also detected in clindamycin and tetracycline. This study confirmed the high susceptibility of *S. uberis* to penicillins used as first-line antimicrobials for *S. uberis* mastitis treatment.

## 1. Introduction

In dairy herds, mastitis is one of the most common diseases with a negative impact on the farming economy [1] related to the treatment of mastitis, reduction of milk production and quality, decreased fertility, and premature culling. 

*Streptococcus uberis* is an environmental pathogen that is responsible for a significant proportion of subclinical and clinical mastitis even in many well-managed herds where the implementation of anti-mastitis measures has been effective in the elimination of contagious pathogens [1,2,3]. The reason for this is the ubiquitous occurrence of this bacterium in the cows’ environment; the teat canal of the mammary gland is thus constantly exposed to contact with this pathogen. In addition, *S. uberis* is a highly genetically variable bacterium that possesses many different virulence factors [3,4]; therefore, gaining control over the emergence of intramammary infections caused by this pathogen represents a major challenge for the management of dairy herds.

Antimicrobial therapy remains the main treatment strategy for mastitis in cattle [5], and the treatment and prevention of this disease result in the frequent use of antimicrobials both in lactating dairy cows as well as during the drying off period. 

Beta-lactams, cephalosporins, lincosamides, and macrolides are most often used to treat *S. uberis* intramammary infection (IMI) [6,7]. In the Czech Republic, intramammary medicinal products containing active substances from the beta-lactam group (narrow-spectrum penicillins, penicillinase-resistant penicillins, and broad-spectrum penicillins, including combinations with beta-lactamase inhibitors) are most often used to treat bovine mastitis. Antimicrobials with indication restrictions are used in smaller quantities, especially cephalosporins of the 3rd and 4th generations. In the Czech Republic, all antimicrobials can be purchased only with a prescription.

The excessive use of antimicrobial agents in dairy herds may lead to increased antimicrobial resistance (AMR) among mastitis pathogens [5]. Recently, many studies have reported the occurrence of phenotypic and genotypic resistance of *S. uberis* isolates to some used antimicrobials, especially tetracycline, clindamycin, erythromycin, and penicillin [8,9,10].

Ideally, the antimicrobial susceptibility of the pathogen causing mastitis should be known when deciding on an antimicrobial treatment. However, treatment must be started as soon as possible, often before the results of susceptibility testing are known. Therefore, it is very important to monitor antimicrobial resistance over time on the farm and region level [11].

This study aimed to determine the antimicrobial susceptibility of *S. uberis* originating from Czech dairy cows with mastitis and to evaluate the development and possible shift in susceptibility during the last five years. We also investigated the spread of resistance genes in the *S. uberis* populations.

## 2. Results

### 2.1. Antimicrobial Susceptibility Testing

**Figure 1:**
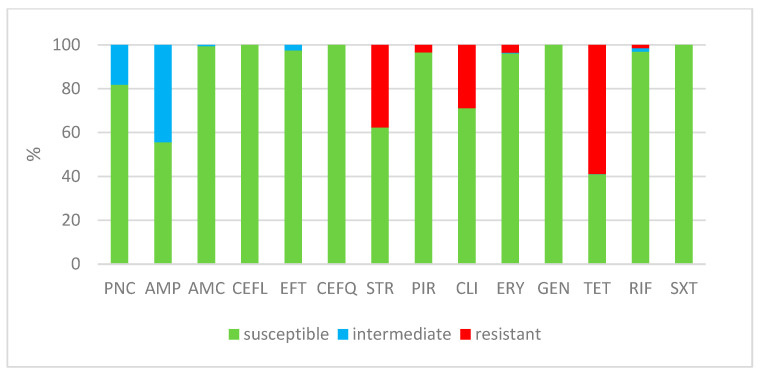
The percentage of susceptible, intermediately resistant, and resistant *S. uberis* isolates from Czech farms in 2019–2023 (n = 667). PNC = Penicillin; AMP = Ampicillin; AMC = Amoxicillin/Clavulanate 2/1; CEFL = Cephalexin; EFT = Ceftiofur; CEFQ = Cefquinome; STR = Streptomycin; PIR = Pirlimycin; CLI = Clindamycin; ERY = Erythromycin; GEN = Gentamicin; TET = Tetracycline; RIF = Rifampin; SXT = Trimethoprin/Sulfamethoxazole 1/19.

**Figure 2:**
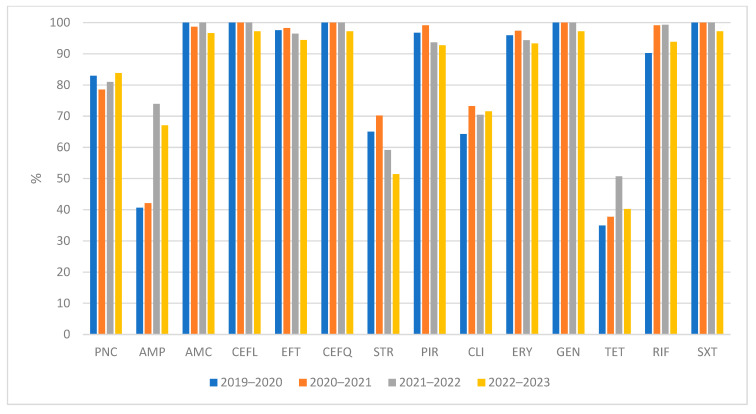
Comparison of the percentages of isolates of *S. uberis* susceptible to the tested antimicrobials in individual years. PNC = Penicillin; AMP = Ampicillin; AMC = Amoxicillin/Clavulanate 2/1; CEFL = Cephalexin; EFT = Ceftiofur; CEFQ = Cefquinome; STR = Streptomycin; PIR = Pirlimycin; CLI = Clindamycin; ERY = Erythromycin; GEN = Gentamicin; TET = Tetracycline; RIF = Rifampin; SXT = Trimethoprin/Sulfamethoxazole 1/19.

Table 1 provides a summary of the results from antimicrobial susceptibility testing conducted on 667 *S. uberis* isolates. It includes the distribution of MICs for various antimicrobials, indicating the number of isolates with corresponding MIC values as specified in the table header. Additionally, Table 1 presents the percentages of isolates categorized as susceptible, intermediately resistant, and resistant, along with the MIC_50_ and MIC_90_ values, both for individual years and cumulatively. Figure 1 illustrates the average percentages of *S. uberis* isolates that were found to be susceptible, intermediately resistant, and resistant among the 667 isolates subjected to testing. A comparison of the occurrence of susceptible isolates in each year is shown in Figure 2. The occurrence of susceptible isolates in individual years was consistent; fluctuations were noted for ampicillin, where the occurrence of susceptible isolates was higher in the last two years, and, on the contrary, for streptomycin a decrease was noted.

*S. uberis* isolates showed resistance mainly to tetracycline, where resistance was recorded in 59% of isolates, followed by streptomycin (38%) and clindamycin (29%). Several isolates were resistant to erythromycin (4%), pirlimycin (3%), and rifampin (1%). A relatively high percentage of intermediately susceptible isolates was recorded for ampicillin (44%) and penicillin (18%). Intermediately susceptible isolates to ceftiofur (3%) were captured. The MIC value for gentamicin did not exceed the high-level aminoglycoside resistance (HLAR) value for any of the isolates. All of the *S. uberis* strains were susceptible to cefquinome, cephalexin, and trimethoprim-sulfamethoxazole. All but one isolate were susceptible to amoxicillin with clavulanic acid (one isolate was moderately susceptible). However, only 28.8% of the isolates were susceptible to all of the tested antimicrobials, and 19.5% of the isolates were multi-resistant (Table 2). The isolates were mostly resistant to tetracycline alone (31.3%); the second most frequent phenotypic profile was co-occurrence of resistance to tetracycline, streptomycin, and clindamycin (16.6%) (Table 2). Approximately 4–5% of strains were resistant only to streptomycin or a combination of streptomycin and clindamycin or tetracycline (Table 2).

### 2.2. Detection of AMR Genes

We screened 140 isolates for the presence of antimicrobial resistance genes; the results are summarized in Table 3. The ant(6)-Ia gene encoding streptomycin resistance was detected in 76 isolates, but only 48 (63%) of them showed phenotypic resistance to streptomycin. Genes lnu(B) and lsa(E), which encode resistance to clindamycin, were detected in 42 isolates, two of which did not show phenotypic resistance. Another gene encoding clindamycin resistance was erm(B), which was detected in six isolates, two of which did not show phenotypic resistance to clindamycin. Phenotypic resistance to erythromycin was observed in nine isolates, but only six harbored the gene encoding its resistance, erm(B).

## 3. Discussion

The objective of this study was to determine the susceptibility and resistance of *S. uberis* isolates to selected antimicrobials and to identify the genes that encode their resistance. The broth microdilution method, which is a quantitative method considered the “gold standard” for susceptibility testing owing to its complexity and accuracy [12], was used. Strains cultured from cows with acute and subclinical mastitis during a five-year study were tested on a plate containing a set of 14 antimicrobials assembled specifically for *S. uberis*. Antimicrobials were selected for the kit with regards to the available intramammary/injectable veterinary medicinal products designed specifically for the treatment of mastitis. For epidemiological reasons, the set also included antimicrobials that are not primarily indicated for the treatment of mastitis, such as ceftiofur, which is not registered in the European Union for the treatment of mastitis but is one of the most commonly used cephalosporins in dairy cattle in the Czech Republic for the treatment of other diseases [13,14]. 

Different genotypes of *S. uberis* are often present in one farm [15]; when isolates with different phenotypic resistance profiles were identified in one farm in our study, all the isolates were included in the evaluation. In many cases, we identified isolates that were highly susceptible to antimicrobials, together with isolates resistant to individual substances or even multi-resistant strains at the same farm. The co-occurrence of strains with different levels of susceptibility in a herd can cause problems in the treatment of mastitis if the causative agent of mastitis is not tested for susceptibility in each individual case, or if we do not have verified susceptibility to antimicrobials in a large number of the *S. uberis* isolates from a given herd within the established bacteriological profile of the herd [16].

Penicillins and the combination of penicillins with penicillinase inhibitors are recommended as first-line drugs because of the high susceptibility of *S. uberis* isolates to these antimicrobials [17,18]. However, over the last two decades a growing number of studies have documented a slow but apparent decline in the susceptibility of streptococci to β-lactams [19]. Decreased susceptibility to penicillin is caused by mutations in genes of the target molecules, known as penicillin-binding proteins (PBPs), resulting in a lower affinity of PBPs for this antimicrobial agent. In vitro, the MIC of penicillin can increase after repeated exposure to gradually increasing concentrations of penicillin in culture media [20].

In our study, resistance to β-lactam antibiotics was very low; no isolate was resistant to them. However, a relatively high incidence of intermediately susceptible isolates was recorded for penicillin (18%) and ampicillin (44%), four isolates (1%) were intermediately susceptible to amoxicillin with clavulanic acid, and seventeen isolates (3%) had a reduced susceptibility to the 3rd generation cephalosporin ceftiofur. The decreased susceptibility to ceftiofur may be due to its extensive use in treating other diseases (especially puerperal metritis and interdigital necrobacillosis). All isolates were susceptible to the cephalosporins, cephalexin (1st generation), and cefquinome (4th generation). For strains with intermediate susceptibility, there is an increased likelihood that the effectiveness of the antimicrobial agent will be reduced in vivo, therapy may fail, or such strains may evolve into resistant strains. Therefore, it is necessary to constantly monitor the development and possible shifts in the susceptibility of isolates, even at the farm level. Even though in other European countries (e.g., Italy and Switzerland) [17,18] the situation is similar to that in the Czech Republic, some studies from distant areas have reported significantly higher levels of resistance. For example, in China [21] and Brazil [22], 31.2% and 50% of isolates were resistant to penicillin, respectively. In Egypt [23], 79.7% were resistant to penicillin and 90% to ampicillin, all isolates were resistant to cefalexin, and 26% were resistant to 3rd generation cephalosporins. The reason for the high level of resistance to many antimicrobials (not only beta-lactams) may be that there is a less strict antibiotic policy in some countries (for example, veterinary antimicrobials are not linked to a medical prescription) [22] and the associated higher consumption of antimicrobials.

The evaluation and comparison of results from different laboratories are difficult because the categorization of strains into susceptible/intermediately susceptible/resistant categories is very imprecise due to the lack of specific interpretation criteria for mastitis strains. Interpretation criteria for mastitis strains of *S. uberis* were provided only for penicillin-novobiocin, cefoperazone, ceftiofur, and pirlimycin. Other cut-off values were derived from values determined for other animals or from human interpretation criteria.

Good susceptibility results were achieved with the macrolide erythromycin (96% of susceptible isolates), which had the lowest MIC_50_ and MIC_90_ values of all of the tested antimicrobials. Similar results were described in Italy, [18] and slightly worse results in Switzerland, [17] where 84.3% of the isolates were susceptible, but the growth of 90% of the isolates was suppressed with concentrations above 4 mg/L (MIC_90_ > 4 mg/L vs. MIC_90_ ≤ 0.06 mg/L in our study). In Brazil, only 53% [22], and in Egypt, only 26% [23] of isolates were susceptible to these substances. However, in our study, 10 isolates with MIC values > 8 mg/L were detected.

The highest susceptibility (100% of highly susceptible isolates) was recorded for trimethoprim in combination with sulfamethoxazole, which is not indicated for the treatment of mastitis, and also for cephalosporins, except ceftiofur. Rifampin (or rifampicin), an ansamycin, also had very good results; in our study, 99% of the strains showed susceptibility. However, the clinical breakpoint value of rifampin for *S. uberis* has not been determined and is derived from the values determined for *S. pneumoniae*. In addition, the European Committee on Antimicrobial Susceptibility Testing recommends not scoring an isolate as “susceptible” (if MIC ≤ 0.125 mg/L), but as “wild type” without mechanisms of resistance to rifampin [24].

When testing the susceptibility of *S. uberis* isolates to lincosamides, we found an increased incidence of strain resistance to clindamycin (29%). For example, a study in Italy in 2021 showed that 82% of isolates were resistant to lincomycin [18], and in Egypt 100% of isolates were resistant [23]. Cross-resistance with macrolides (for example, erythromycin) has been described for lincosamides [25]. In our study, of the 193 isolates resistant to clindamycin, only nine were simultaneously resistant to erythromycin. Other isolates showed the L-phenotype of lincosamide resistance (that is, a lincosamide-resistant phenotype associated with macrolide susceptibility). 

In the group of lincosamides, pirlimycin (a more effective derivative of clindamycin) is more often used to treat mastitis; the recommended and approved 8-day treatment (eight doses every 24 h) has very good results in the form of clinical but also bacteriological cure of *S. uberis* infections. In addition, pirlimycin is the only antimicrobial among our set of 14 antimicrobials for which a cut-off value has been determined specifically for mastitis *S. uberis* isolates; therefore, we can classify the isolate as susceptible or resistant according to the MIC value with greater certainty. In our study, only 3% of the isolates were resistant to this antimicrobial. Although pirlimycin is used for the treatment of *S. uberis* mastitis and has one of the few defined interpretation criteria directly for mastitis isolates, in most published studies involving *S. uberis* resistance to antimicrobials, only lincomycin or clindamycin were tested in a group of lincosamides. Therefore, there are relatively few data on the susceptibility of pirlimycin compared to other antimicrobials. Studies that included pirlimycin in their testing revealed significantly more resistant strains compared to our results; for example, 11.8% in Switzerland [17] and approximately 40% of resistant isolates in Brazil [22].

Streptococci are naturally resistant to aminoglycosides due to the reduced permeability of the cell wall to these substances; therefore, a high MIC_50_ and MIC_90_ is always detected (in our study for streptomycin MIC_50_ = 256 mg/L and MIC_90_ > 512 mg/L). Therefore, aminoglycosides alone are unsuitable for the treatment of streptococcal infections; however, they can be used in combination with antimicrobials that disrupt the cell wall (for example, beta-lactams). This combination is not effective enough for strains with high resistance to aminoglycosides (HLAR positive—high-level aminoglycoside resistance), which are defined by a high MIC value for gentamicin (MIC > 128 mg/L). In our study, no isolate showed high resistance.

Another antimicrobial for which a long-term high MIC_90_ value has been recorded is tetracycline (MIC_90_ > 32). In the Czech Republic, we detected 59% of isolates resistant to tetracycline. A high incidence was also recorded in other countries such as Thailand (82%) [9], Egypt (65%) [23], China (59%) [26], Germany (42%) [27], and Canada (38.6%) [28]. This is probably due to the long-term massive use of tetracyclines to treat various infections, as tetracyclines are slowly degraded in the environment and slowly eliminated from the body after application, thus leading to high exposure. All these factors contribute to increased selective pressure for tetracycline resistance in bacteria [9,29]. Although tetracycline is not routinely used to treat mastitis, the resistance of *S. uberis* to tetracycline is not surprising. Conversely, in some countries, such as Sweden, where tetracyclines were used less often than penicillins, there was a significantly lower percentage of tetracycline-resistant strains (12%) [30].

Overall, we found that 28.8% of isolates were susceptible to all of the tested antimicrobials and 19.5% of isolates were multi-resistant, that is, resistant to three or more groups of antimicrobials. The most frequent isolates were resistant to tetracycline alone (31.3%) and the second most common phenotypic resistance profile was combined resistance to tetracycline, streptomycin, and clindamycin (16.6%). A similar rate of multi-resistant strains was found in Italy (25.4%) [18]; however, 100% of multi-resistant streptococci was recorded in China [31] and Egypt [23]. These differences between countries may be due to different levels of control over the use and consumption of antimicrobials, allowing for more massive and often unjustified use of antimicrobials in some countries. These differences could also be caused by the use of different testing methods (disc diffusion method vs. broth dilution method vs. E-test) and different interpretation criteria. Care should be taken when interpreting and comparing the AMR results because there are no interpretive criteria for most antimicrobials to precisely categorize *S. uberis* isolates. It is difficult to accurately assess the level of antimicrobial resistance in mastitis pathogens using only cut-off values (distinguishing wild-type strains without resistance mechanisms from non-wild-type strains) or breakpoint values (breakpoint values of susceptibility/resistance determined on the basis of clinical response), often taken from other animal species, other groups of bacteria, or standards of human medicine. Therefore, it is more accurate to compare MIC_50_ and MIC_90_ values, which provide useful information about the level of resistance and these values should be reported whenever antimicrobial susceptibility and resistance data are analyzed.

The genomes of the isolates were screened for AMR genes and then compared with phenotypic resistance. In general, antibiotic resistance gene prediction does not always match phenotypic results [10,32]. Some discrepancies were also observed in our study. In streptomycin evaluation, 76 isolates harbored the AMR gene, but 28 of them (36.8%) did not show phenotypic resistance. A similar situation was observed for clindamycin in four (8.5%) isolates and for tetracycline in one isolate (1.2%). This was probably due to a lack of gene expression, possibly due to the presence of silent genes. Silent AMR genes are a common phenomenon in many bacteria, with an occurrence rate ranging from 0.16% to 79.31% [32]. Genes that remain silent might get triggered into activity through mutations or recombination. Furthermore, when these genes are transferred to a new host via horizontal gene transfer [32] they can potentially become active. There is a viewpoint held by some researchers suggesting that numerous genes remain inactive in laboratory settings but are typically expressed in their natural environment. This is because laboratory conditions represent an approximation of the natural environment and can thereby influence the bacterial phenotype. Monitoring AMR genes, including silent genes, is necessary to estimate the antimicrobial resistance potential of a bacterial population [32].

Three isolates categorized according to CLSI as resistant to erythromycin did not carry the *erm(B)* gene encoding this resistance. Here, we probably encountered a problem with the interpretation criteria because all of these isolates showed MIC = 1 mg/L, which is categorized as resistant in CLSI [33] (where the interpretation criteria for erythromycin are derived from human criteria), whereas, according to CASFM-VET 2021 [34], isolates would be categorized as resistant only from an MIC value above 4 mg/L, which in our study would correspond much more to the occurrence of the AMR gene. A slightly different situation was noted with rifaximin, where two resistant isolates had a significantly higher MIC value (>4 mg/L) than the rest of the population; however, no gene encoding this resistance was detected, probably due to the insufficiency of the AMR database.

## 4. Materials and Methods

In total, 952 *S. uberis* strains were isolated from milk samples of 216 herds between 2019–2023. All of the strains were tested for susceptibility to 14 antimicrobials. Strains that originated from the same herd and showed the same susceptibility to antimicrobials were excluded from the study; thus, 667 strains were evaluated. Parts of them were randomly selected (n = 140) for the detection of resistance genes using the whole-genome sequencing method (WGS).

### 4.1. Bacterial Sampling

In this study, we included 667 strains of *S. uberis*, which were obtained from cases of both subclinical mastitis (identified through elevated somatic cell counts exceeding 400,000 cells/mL in production control programs) and clinical mastitis (characterized by abnormal milk and udder changes like swelling, pain, or heat) in cows. These strains were sourced from samples collected across 216 farms located in various regions of the Czech Republic over a period spanning from May 2019 to April 2023. Upon collection, milk samples were stored in containers at temperatures ranging from 6 °C to 8 °C and were promptly transported to the laboratory within 4 h. Alternatively, some samples were frozen at −18 °C and delivered within one week. In cases where multiple *S. uberis* isolates were obtained from a single farm within a given year, all isolates with distinct susceptibility profiles were included in the study.

### 4.2. Bacterial Isolation and Identification

Ten microliters of milk samples were inoculated onto Columbia agar plates (Oxoid, Basingstoke, UK) containing 5% defibrinated sheep blood and incubated at 37 °C for 24 h. The isolated bacteria were initially identified based on their colony characteristics and further confirmed using a phenotypic molecular approach involving MALDI-TOF-MS mass spectrometry (Bruker Daltonics GmbH, Bremen, Germany). Additionally, the strains were verified through the detection of the *S. uberis*-specific 16S rRNA gene using polymerase chain reaction (PCR) (see Table 1 for details). If *S. uberis* was cultured in the sample, it was always included in the study, regardless of the possible simultaneous occurrence of other pathogens. If more than 3 morphologically different colonies grew on the plate, the sample was discarded as contaminated.

### 4.3. Antimicrobial Susceptibility Testing

We conducted antimicrobial susceptibility testing (AST) for 14 selected antimicrobials by determining their minimum inhibitory concentrations (MICs) using the microdilution method, following internationally recognized protocols established by the Clinical and Laboratory Standards Institute [33,35]. The MICs were determined using diagnostic kits developed by our co-authors at the Veterinary Research Institute in Brno, Czech Republic. The growth medium used for diluting the antimicrobials consisted of Mueller Hinton Broth (BD Difco, Franklin Lakes, UK) supplemented with 4% Lysed Horse Blood (Labmediaservis, Jaromer, Czech Republic). To ensure the accuracy of our examination, we concurrently assessed a control reference strain, Streptococcus pneumoniae ATCC 49,619 [33]. The tested antimicrobials used for AST (Discovery Fine Chemicals Limited, Wimborne, UK) represented nine antimicrobial groups: lincosamides (clindamycin, pirlimycin), aminoglycosides (gentamicin, streptomycin), macrolides (erythromycin), sulfonamides (sulfamethoxazole with trimethoprim), tetracyclines (tetracycline), ansamycins (rifampin), and three categories of penicillins (1) narrow and broad-spectrum, penicillinase-sensitive (penicillin, ampicillin), (2) penicillins with beta-lactamase inhibitors (amoxicillin with clavulanic acid), and (3) cephalosporins (cephalexin–1st generation, ceftiofur–3rd generation, and cefquinome–4th generation). We categorized the isolates as susceptible, intermediate, or resistant based on clinical breakpoints defined in the CLSI documents [33], the European Committee on Antimicrobial Susceptibility Testing’s Breakpoint tables for bacteria [24], and the Comité de l’Antibiogramme de la Société Francaise de Microbiologie’s Veterinary Recommendations for 2021 [34]. An isolate was considered multidrug resistant if it displayed resistance to at least one substance from three or more antimicrobial groups [36].

### 4.4. Detection of AMR Genes

One hundred and forty isolates from 74 farms collected between 2019 and 2022 were randomly selected for whole-genome sequencing (WGS) to detect the presence of antimicrobial resistance genes. 

#### 4.4.1. Nucleic Acid Extraction

The extraction of Deoxyribonucleic acid (DNA) was carried out employing the QIAamp DNA Mini Kit (Qiagen Inc., Canada). Initially, a 1.5 mL volume of the bacterial culture suspension was subjected to centrifugation at 5000× *g* for 10 min, resulting in the formation of a bacterial pellet. This pellet was then resuspended in 180 µL of a lysozyme lysis buffer, which consisted of 20 mg/mL lysozyme, 20 mM Tris–HCl at pH 8.0, 2 mM EDTA, and 1.2% Triton. The mixture was subsequently incubated at 37 °C for 30 min. Following this incubation, 20 µL of ProtK from the Qiagen Kit, 200 µL of buffer AL (Qiagen Kit), and 1 µL of RNase A from PureLink (Invitrogen Thermo Fisher Scientific, Waltham, MA, USA) were introduced into each tube and thoroughly mixed by vortexing. The samples were then incubated at 56 °C for 30 min, followed by a final incubation at 70 °C for 5 min. To this mixture, 200 µL of ethanol (95–100%) was added and the solution was pulse vortexed for 15 s. Subsequently, the samples were loaded onto QIAamp mini spin columns and the extraction process was continued following the instructions provided in the kit. In the final step, all samples were eluted with 80 µL of UltraPure Water (Invitrogen, Waltham, MA, USA). To assess DNA quality, determine DNA concentration, and calculate the 260:280 ratios, 1 µL of each DNA sample was utilized with the NanoDrop 1000 spectrophotometer (Thermo Scientific, Wilmington, DE, USA).

#### 4.4.2. Whole-Genome Sequencing 

The sequencing library was prepared according to the manufacturer’s protocol (Nextera XT DNA Sample Prep Kit, Illumina, Inc. San Diego, CA, USA) using 1 ng of DNA per sample. The DNA concentration of the samples was measured using a Qubit fluorometer (Thermo Fisher Scientific, Waltham, MA, USA). The Illumina NextSeq^®^ 500/550 High Output Kit v2 (Illumina, San Diego, CA, USA) and the NextSeq 500 instrument (Illumina, San Diego, CA, USA) were utilized for paired 2 × 150 bp whole-genome sequencing. Raw reads were processed using the TORMES 1.3.0 pipeline [37]. Assembly was conducted using the SPAdes assembler [38] with default settings. The processing was parallelized using GNU Parallel [39]. Taxonomic identification was performed by Kraken2 [40]. Antibiotic resistance genes were identified by screening genomes against the Resfinder [41], CARD [42], and ARG-ANNOT [43] databases using Abricate [44] within the TORMES 1.3.0 pipeline.

## 5. Conclusions

Antimicrobials are a sufficiently effective and irreplaceable tool for the control and treatment of infections. However, they must be used after careful consideration. Antimicrobials should not be used as a cover for deficiencies in hygiene, nutrition, care for the physiological needs of animals, and animal handling. In our study, the validity of using penicillins as first-line antimicrobials for mastitis caused by *S. uberis* was confirmed, as all isolates were susceptible to these substances. However, in some countries a high percentage of resistant isolates have been recorded. The different susceptibility values of the strains in published studies may be due to the antibiotic policy of the given country and the related consumption of different groups of antimicrobials. However, the comparison of the results can be distorted by the use of different test methods, and the lack of specific interpretation criteria for mastitis pathogens can cause further inaccuracies in the categorization of isolates into susceptible or resistant. The introduction of additional veterinary clinical breakpoints can lead to improved antibiotic surveillance and optimization of the treatment of mastitis and other animal diseases.

## Figures and Tables

**Table 1:** MICs’ distribution for antimicrobials—percentages of resistant isolates, MIC_50_ and MIC_90_ values in *S. uberis* isolates from Czech farms in 2019–2023.

	MIC (mg/L)																R	MIC_50_	MIC_90_
	0.03	0.06	0.13	0.25	0.5	1	2	4	8	16	32	64	128	256	512	1024	(%)	(mg/L)	(mg/L)
**May 2019–April 2020: n = 123 (from 72 farms)**																			
PNC		27	12	63	21												0	0.25	0.5
AMP			11	17	22	73											0	1	1
AMC	24	4	6	83	6												0	0.25	0.25
CEFL							123										0	≤2	≤2
CEF						103	17	3									0	≤1	2
CEFQ					123												0	≤0.5	≤0.5
STR												15	29	36	2	41	35	256	>512
PIR					80	25	14	3	1								3	≤0.5	2
CLI		76	1	2		1	15	27		1							36	≤0.06	4
ERY		117		1	1		1	2	1								3	≤0.06	≤0.06
GEN								6	19	23	75						0	32	32
TET				42		1			1	15	28	36					65	32	>32
RIF	24	87	10						2								2	0.06	0.06
SXT			123														0	≤0.125	≤0.125
**May 2020–April 2021: n= 228 (from 119 farms)**																			
PNC		45		134	48	1											0	0.25	0.5
AMP			15	31	50	124	8										0	1	1
AMC	23	23	7	106	66	3											0	0.25	0.5
CEFL							225	3									0	≤2	≤2
CEF						146	78	4									0	≤1	2
CEFQ					225	3											0	≤0.5	≤0.5
STR												48	71	41	15	53	29	128	>512
PIR					173	30	23	1	1								1	≤0.5	2
CLI		164	3			1	15	43		2							27	≤0.06	4
ERY		221	1		1	1	1	1		2							3	≤0.06	≤0.06
GEN								11	18	57	138	4					0	32	32
TET				84	1	1			1	21	50	70					62	32	>32
RIF	9	178	39						2								1	0.06	0.125
SXT			227	1													0	≤0.125	≤0.125
**May 2021–April 2022: n = 142 (from 75 farms)**																			
PNC		27	11	77	27												0	0.25	0.5
AMP		18	10	60	17	36	1										0	0.25	1
AMC	20	7	10	88	17												0	0.25	0.5
CEFL							141	1									0	≤2	≤2
EFT						111	26	5									0	≤1	2
CEFQ					140	2											0	≤0.5	≤0.5
STR												21	27	36	22	36	41	256	>512
PIR					104	18	11	7	2								6	≤0.5	2
CLI		95	4	1		1	13	19	7	2							30	≤0.06	4
ERY		126	8			4	1	1		2							6	≤0.06	0.125
GEN								7	11	48	62	7	7				0	32	32
TET				72				1	1	16	26	26					49	≤0.25	>32
RIF	26	92	23						1								1	0.06	0.125
SXT			142														0	≤0.125	≤0.125
**May 2022–April 2023: n= 174 (from 94 farms)**																			
PNC		39	9	102	23		1										0	0.25	0.5
AMP		14	13	60	33	52	2										0	0.25	1
AMC	36	4	34	87	12	1											0	0.25	0.25
CEFL							173		1								0	≤2	≤2
EFT						129	40	5									0	≤1	2
CEFQ					171	3											0	≤0.5	≤0.5
STR												53	11	28	6	76	46	256	>512
PIR					130	21	15	4	4								4	≤0.5	2
CLI		124	3	1			14	27	2	3							26	≤0.06	4
ERY		165	2			1				6							4	≤0.06	≤0.06
GEN								6	28	52	74	14					0	32	32
TET				70		2			2	27	31	42					57	16	>32
RIF	43	93	32	1					5								3	0.06	0.125
SXT			173	1													0	≤0.125	≤0.125
**May 2019–April 2023: n = 667 (from 216 farms)**																			
PNC		138	32	376	119	1	1			0							0	0.25	0.5
AMP		32	49	168	122	285	11			0							0	0.5	1
AMC	103	38	57	364	101	4			0								0	0.25	0.5
CEFL							662	4	1						0		0	≤2	≤2
EFT						489	161	17		0							0	≤1	2
CEFQ					659	8											0	≤0.5	≤0.5
STR												137	138	141	45	206	38	256	>512
PIR					487	94	63	15	8								3	≤0.5	2
CLI		459	11	4	0	3	57	116	9	8							29	≤0.06	4
ERY		629	11	1	2	6	3	4	1	10							4	≤0.06	≤0.06
GEN								30	76	180	349	25	7				0	32	32
TET				268	1	4	0	1	5	79	135	174					59	16	>32
RIF	102	450	104	1				0	10								1	0.06	0.125
SXT			665	2													0	≤0.125	≤0.125

PNC = Penicillin; AMP = Ampicillin; AMC = Amoxicillin/Clavulanate 2/1; CEFL = Cephalexin; EFT = Ceftiofur; CEFQ = Cefquinome; STR = Streptomycin; PIR = Pirlimycin; CLI = Clindamycin; ERY = Erythromycin; GEN = Gentamicin; TET = Tetracycline; RIF = Rifampin; SXT = Trimethoprim/Sulfamethoxazole 1/19; MIC = Minimal Inhibitory Concentration; The tested dilution ranges of individual antimicrobials are delimited by the grey zone. The numbers in the colored areas indicate the numbers of isolates with their corresponding MIC value (mg/L). Sensitive isolates are marked in green, intermediate isolates in blue, and resistant isolates in orange. Numbers in the grey zone indicate the number of isolates with MIC values higher than tested dilution range. The MIC_50_ and MIC_90_ values represent the lowest concentration (mg/L) inhibiting the growth of 50% and 90% of the isolates in the bacterial culture with a density of 10^5^ CFU/mL. (Interpretation criteria for categorization of the isolates as susceptible/intermediate/resistant were determined according to EUCAST 2022, CASFM-VET 2021, and CLSI 2020-VET01S-Ed5).

**Table 2:** Resistance profiles for *S. uberis* isolates from Czech farms in 2019–2023 (n = 667).

No. of Antimicrobial Groups	Phenotype Profile of Resistance	Percentage of Resistant Isolates
0	susceptible	28.8
1	TET	31.3
1	STR	4.3
1	PIR	0.3
1	CLI	0.6
1	ERY	0.7
2	STR, CLI	4.8
2	CLI, TET	1.0
2	STR, TET	5.1
2	ERY, TET	1.2
2	TET, RIF	0.9
2	STR, PIR, CLI	1.3
3	STR, CLI, TET	16.6
3	STR, TET, RIF	0.1
3	STR, CLI, RIF	0.1
3	STR, ERY, TET	0.3
3	CLI, ERY, TET	0.1
3	STR, PIR, CLI, TET	0.6
3	PIR, CLI, ERY, TET	0.3
4	STR, CLI, ERY, TET	0.1
4	STR, CLI, TET, RIF	0.1
4	STR, PIR, CLI, ERY, TET	0.7
4	STR, PIR, CLI, TET, RIF	0.1
Multi-resistant isolates		19.5

STR = Streptomycin; PIR = Pirlimycin; CLI = Clindamycin; ERY = Erythromycin; TET = Tetracycline; RIF = Rifampin.

**Table 3:** Screening for antimicrobial resistance genes in *S. uberis* isolates (n = 140) and comparison to phenotypic resistance.

Substance	Genes Detected	No. of Isolates with AMR Gene	No. of Isolates Phenotypic Resistant	Gene+/Phen−	Gene−/Phen+
streptomycin	*ant(6)-Ia*	76	48	28	0
clindamycin	*lnu(B) + lsa(E); erm(B)*	47	43	4	0
pirlimycin	*lnu(B) + lsa(E); erm(B)*	9	9	0	0
tetracyklin	*tet(M); tet(L); tet(O); tet(S)*	81	80	1	0
erythromycin	*erm(B)*	6	9	0	3
rifaximin	-	0	2	0	2

AMR = Antimicrobial resistance; gene+/phen− = Number of isolates possessing the AMR gene, but did not show phenotypic resistance to substance; gene−/phen+ = Number of isolates phenotypic resistant to substance, but with no AMR gene detected.

## Data Availability

Raw data supporting the conclusions of this study are available from the authors upon request.
